# Adaptation and Survival of *Burkholderia cepacia* and *B. contaminans* During Long-Term Incubation in Saline Solutions Containing Benzalkonium Chloride

**DOI:** 10.3389/fbioe.2020.00630

**Published:** 2020-06-26

**Authors:** Mariana Tavares, Mariya Kozak, Alexandra Balola, Carla P. Coutinho, Cláudia P. Godinho, A. Amir Hassan, Vaughn S. Cooper, Isabel Sá-Correia

**Affiliations:** ^1^iBB - Institute for Bioengineering and Biosciences, Instituto Superior Técnico, Universidade de Lisboa, Lisbon, Portugal; ^2^Department of Bioengineering, Instituto Superior Técnico, Universidade de Lisboa, Lisbon, Portugal; ^3^Department of Microbiology and Molecular Genetics, University of Pittsburgh School of Medicine, Pittsburgh, PA, United States

**Keywords:** *Burkholderia cepacia* complex, *Burkholderia cepacia*, *Burkholderia contaminans*, pharmaceutical products' contamination, nutrient starvation adaptation, benzalkonium chloride adaptation, small colony variants

## Abstract

The *Burkholderia cepacia* complex (Bcc) is a group of opportunistic pathogenic bacteria with a remarkable metabolic capacity and broad genotypic/phenotypic plasticity, allowing their adaptation to hostile conditions, including nutrient depleted solutions containing antimicrobial agents. Bcc bacteria are feared contaminants in pharmaceutical industries and cause nosocomial outbreaks, posing health threats to immunocompromised individuals and cystic fibrosis (CF) patients. In this study, the adaptation and survival of *B. cepacia* and *B. contaminans* isolates was investigated after long-term incubation in nutrient depleted saline solutions supplemented with increasing concentrations of the biocidal preservative benzalkonium chloride (BZK), recreating the storage conditions of pharmaceutical products. These epidemiologically related isolates were recovered from intrinsically contaminated saline solutions for nasal application and from two CF patients. Long-term incubation in saline solutions containing BZK led to the development of bacterial sub-populations that survived for at least 16 months, despite an initial 2–3 log decrease in viability, displaying a progressive dose-dependent decrease of colony and cell size, including the appearance of small colony variants (SCVs). Bacterial colonies lost pigmentation, changed the morphotype from rough to smooth and produced more spherical cells during extended incubation with BZK. The development of macroscopically visible cellular aggregates, rich in polysaccharide and harboring viable cells in their interior was triggered by BZK. The existence of a metabolic pathway for BZK degradation was confirmed through genome analysis. This study reveals mechanisms underlying the prevalence of Bcc bacteria as contaminants of pharmaceutical products containing BZK, which often lead to false-negative results during quality control and routine testing.

## Introduction

The *Burkholderia cepacia* complex (Bcc) is a group of Gram-negative β-proteobacteria with an ubiquitous environmental distribution (Compant et al., 2008), that have emerged as human opportunistic pathogens able to cause severe infections in patients with underlying disease, namely cystic fibrosis (CF) and chronic granulomatous (Mahenthiralingam et al., 2005; Eberl and Vandamme, 2016), and in immunocompromised individuals. Chronic Bcc infections are very difficult to eradicate, mainly due to their intrinsic resistance to a large number of antimicrobials and their ability to adapt to the adverse conditions that characterize the human host (Coutinho et al., 2011b). Bcc bacteria are well-known for their ability to remain viable for prolonged periods of time in hostile conditions (Torbeck et al., 2011), being commonly associated with contamination of water-based environments, solutions with low nutrient concentrations and pharmaceutical products containing antimicrobial agents (disinfectant solutions, hospital soaps, nasal sprays, mouth wash, and anesthetics) (Sutton and Jimenez, 2012; Jimenez et al., 2015; Ali, 2016). Numerous reports of Bcc-related nosocomial outbreaks have been published in the last decades (Tavares et al., 2020), and multiple products have been recalled from the market (United States Food Drug Administration, 2019), due to contamination with this group of bacteria, highlighting the potential public health threat imposed by these objectionable microorganisms (Mahenthiralingam et al., 2005; Sutton and Jimenez, 2012).

Multiple contamination outbreaks have been linked, in particular, to the biocide benzalkonium chloride (BZK), a cationic quaternary ammonium compound that exerts its effects by compromising the structure and stability of the cytoplasmic membrane (McDonnell and Russell, 1999; Weber et al., 2007; Fazlara and Ekhtelat, 2012). This biocide is included in a variety of commercially available products' formulation, at concentrations ranging from 200 to 50,000 μg/mL (Kim et al., 2015). Several reports and studies have demonstrated that biocides, when present in low concentrations (referred to as preservatives), do not prevent contamination, since some Bcc strains are able to persist in preserved solutions (Avgeri et al., 2009; Rose et al., 2009; Torbeck et al., 2011; Buffet-Bataillon et al., 2012; Ahn et al., 2016, 2017). The best described Bcc species in terms of BZK resistance is *B. cenocepacia* AU1054, the strain used by Ahn et al. (2016) to perform transcriptomic studies that allowed the identification of two main mechanisms responsible for BZK resistance (efflux activity and metabolic inactivation through biodegradation), as well as a putative metabolic pathway responsible for its degradation (Ahn et al., 2016).

When exposed to stressful conditions, Bcc populations can undergo phenotypical and genetic adaptations that confer selective advantage. One example of such versatility is the formation of small colony variants (SCVs), which typically exhibit one-tenth the size of the colonies associated with the parent strain, and are characterized by a slow specific growth rate (Häussler et al., 1999; Proctor et al., 2006; Kahl, 2014; Bui and Kidd, 2015). Besides being considered a pathogenesis factor, formation of SCVs is also thought to contribute for the establishment of recurrent infections, leading to lung function deterioration and development of fatal systemic infections (Häussler et al., 2003; Proctor et al., 2006). As SCVs require prolonged incubation periods for their manifestation, they are mostly overgrown by normal-sized colonies, making their isolation and identification a difficult task (Torbeck et al., 2011; Jimenez et al., 2015), which can lead to false-negative results during quality control and routine testing during pharmaceutical manufacturing (Jimenez et al., 2015). The undetected presence of Bcc bacteria constitutes a serious healthcare problem, especially if these contaminated products are used by susceptible patients, negatively affecting their treatment options and general prognosis.

An unusually high frequency of *B. cepacia* infections was detected among the Portuguese CF community, in the period between 1995 and 2005, affecting as much as 85% of the CF patients under surveillance at the major Portuguese CF center at Hospital de Santa Maria (HSM) in Lisbon (Cunha et al., 2003, 2007). A routine market surveillance analysis performed by INFARMED, the Portuguese Medicines and Health Products Authority, in 2003 and 2006, revealed that several batches of non-sterile saline solutions used for nasal application, produced by two local manufacturers, greatly exceeded the microbiological quality limits established by European Pharmacopoeia VII, and *B. cepacia* bacteria were subsequently isolated from those products (Cunha et al., 2007). Further analysis confirmed that the clinical clones obtained from CF patients and the strains detected in saline solutions were indistinguishable (Cunha et al., 2007). When this outbreak occurred, all of the clones identified by our laboratory were classified as *B. cepacia*. In 2015, a re-examination of the isolates was conducted and 20 out of 56 were reclassified as *B. contaminans* (Coutinho et al., 2015). The epidemiological analysis carried out by our laboratory identified *B. cepacia* and *B. contaminans* as emerging Bcc species associated with CF infections (Cunha et al., 2003, 2007; Correia et al., 2008; Coutinho et al., 2011b), since the overall prevalence of these two species in CF patients is low (Moreira et al., 2017).

In the present study, a set of epidemiologically related *B. cepacia* and *B. contaminans* isolates, recovered from contaminated saline solutions and from sputum samples of CF patients, were used to examine the adaptation and survival of these bacteria during long-term incubation, at 23°C in the dark, in artificially contaminated saline solutions deprived of any nutrient source and supplemented with increasing BZK concentrations. These conditions were used to mimic the normal storage conditions of saline solutions for nasal application from which the Bcc isolates were obtained. A number of phenotypic characteristics were assessed in regular intervals of time, up to 16 months, including cellular viability, cell/colony diameter and morphology, as well as pigmentation changes and the formation of cellular aggregates and their characterization in terms of polysaccharide vs. protein content.

## Materials and Methods

### Bacterial Isolates and Culture Conditions

Five *B. cepacia* clonal isolates were analyzed in this study, including two that were recovered from the contaminated saline solutions detected by INFARMED and three collected from a CF patient (patient AL) (Cunha et al., 2007). Of the four *B. contaminans* clonal isolates analyzed, one was isolated from contaminated saline solutions, in 2003, and three were obtained from another CF patient (patient V) (Cunha et al., 2007) (Table 1). Previous studies showed that the *B. cepacia* and *B. contaminans* isolates recovered from saline solutions were indistinguishable from those isolated from CF patients, suggesting that they were epidemiologically related (Cunha et al., 2007; Coutinho et al., 2015). These clinical isolates were recovered, as part of the hospital routine, from the sputum of CF patients under surveillance at Hospital de Santa Maria, Centro Hospitalar Lisboa Norte (CHLN) EPE. Studies involving these isolates were approved by the CHLN ethics committee and the anonymity of the patients was preserved. Informed consent was also obtained from all participants and/or their legal guardians. All the methods were performed in accordance with the relevant guidelines and regulations. Bacterial cultures were stored at −80°C in 1:1 (vol/vol) glycerol. When in use, bacteria were cultivated in *Pseudomonas* isolation agar (PIA; Difco) plates. The isolates used in the present study are listed in Table 1.

**Table 1:** List of *B. cepacia* and *B. contaminans* clonal isolates used in this study, obtained from contaminated saline solutions or sputum samples from CF patients.

**Species**	**Isolate source**	**Isolate ID**	**Isolation date**	***recA* RFLP profile**	**Ribopattern**	**References**
*B. cepacia*	Saline solution	IST612	November-December 2003	D	19	Cunha et al., 2007; Coutinho et al., 2015
		IST701	March 2006	D	19	
	Patient AL	IST4152	30/10/2003	D	19	
		IST4168	18/5/2004	D	19	
		IST4222	14/12/2005			
*B. contaminans*	Saline solution	IST601	December 2003	E	17	
	Patient V	IST4148	03/09/2003	E	17	
		IST4241	28/06/2004	E	17	
		IST4224	27/12/2005	E	17	

### Long Term Adaptation Assays to Benzalkonium-Supplemented Saline Solutions

Liquid cultures of the Bcc isolates were grown in Luria-Bertani (LB; NZYtech) medium (37°C, 250 rpm) until mid-exponential phase and centrifuged (10 min, 7,000 rpm and 20°C), washed twice with sterile NaCl 0.9%, and adjusted to a standardized culture OD_640nm_ of 0.2 in 150 mL of NaCl 0.9%. The resulting cellular suspension was used to inoculate glass flasks containing **(i)** only NaCl 0.9%, **(ii)** NaCl 0.9% supplemented with 0.0053% (w/v) of BZK (SIGMA-ALDRICH) and **(iii)** NaCl 0.9% supplemented with 0.05% (w/v) of BZK. The different BZK concentrations were chosen on the basis of the expected effects each would have in the bacterial populations. Since sub-minimum inhibitory concentrations of BZK ranging from 10 to 50 μg/mL have been reported to affect bacterial survival without completely killing the population (Kim et al., 2015), a concentration of 0.0053% (w/v) was used to guarantee a detectable effect in the populations' survival. A 10x higher BZK concentration (0.05% (w/v)) was also tested to evaluate the effects of a presumably lethal biocide concentration. Flasks were incubated at 23°C, in the dark, without agitation, during the entire course of the experiment (16 months), to replicate the typical storage conditions employed in the pharmaceutical industry for this type of products.

### Cell Viability Analysis

Bacterial samples were harvested from the glass flasks representing each incubation condition, after gently homogenizing the content, and, after serial dilution in NaCl 0.9%, with strong vortex agitation, they were plated onto Tryptic Soy Agar medium (TSA; Difco). This solid growth medium was used because it was found to be the most appropriate to distinguish different colony morphologies. Plates were incubated at 30°C for 72 h in order to allow a clearer observation of the size differences between normal and small colony variants (SCVs) and other morphotypes. Samples collected from the flasks containing BZK were filtered, after serial dilution, using 0.2 μm pore size nitrocellulose membrane filters (Whatman) to avoid cell growth inhibition by BZK. Cell viability assessment was performed using conventional methods, based on colony forming units (CFU), by estimating the number of viable bacteria that can form colonies on an agar plate.

### Colony Size and Morphology Assessment

Colony morphology analysis was performed using a stereomicroscope and a Kl 2500 LCD cold light source (Zeiss). The images were captured with an Axicam 503 color camera (ZEISS) and colony diameters were determined using the ZEN 2.3 software. At least 10 images were captured for each sample. Approximately 100 colonies of each bacterial isolate were randomly selected for diameter measurement.

### Cell Size and Morphology Assessment

Cell size and morphological parameters, such as length and width, were determined by fluorescence microscopy. Cells were harvested and stained with the LIVE/DEAD® *Bac*Light^TM^ Bacterial Viability Kit from Molecular Probes (Invitrogen Co., Spain). Briefly, 5 mL of an overnight culture grown in liquid LB at 37°C, 250 rpm, were washed with NaCl 0.9% (centrifuged twice for 10 min at 7,000 rpm, 21°C) and resuspended in 4 mL of NaCl 0.9%. From these cell suspensions, 30 μL were mixed with 0.2 μL of each fluorophore (SYTO 9 and Propidium iodide, each diluted 1:18 from the original stock) and left to incubate at room temperature in the dark for 5 min. Cells were observed using a ZEISS Axioplan fluorescence microscope with an HBO 50 burner and a HAL 100 halogen illuminator and a filter with 450–490 nm excitation and 520 nm emission. Images were captured with a microscope camera (ZEISS Axicam 503 color) using ZEN 2.3 (ZEISS) Imaging software. Image analysis was carried out using ImageJ 1.52a. At least 6 images were taken for each sample. Surface area was calculated as 4πr^2^ + 2πL and volume as 4/3 πr^3^ + πr^2^L, where r represents radius or width/2, and L represents the length of the cell.

### Benzalkonium Susceptibility Assays (MIC and MBC Determination)

To determine the susceptibilities to BZK (SIGMA-ALDRICH) displayed by the isolates under study, a modified broth minimum inhibitory concentration (MIC) method was performed, using 96-well polystyrene microplates (Greiner Bio-One, Germany). Cell cultures grown in liquid LB (37°C, 250 rpm) until mid-exponential phase were centrifuged (10 min, 7,000 rpm, 20°C), resuspended in Mueller-Hinton (MH; Fluka Analytical) broth, and adjusted to a standardized OD_640_ of 0.011. Of these cell cultures, 190 μL were used to inoculate the wells of 96-well microplates, together with 10 μL of BZK solutions at final concentrations of 16, 32, 64, 128, 192, 256, 384, and 512 μg/mL. The 96-well microplates were incubated at 23°C, in the dark, for 72 h. The lowest BZK concentration sufficient to inhibit bacterial growth was defined as the MIC or susceptible concentration for each Bcc isolate. These tests were performed in three biologically independent experiments.

The minimum bactericidal concentration (MBC) was also determined. After the above described 72 h incubation period, 4 μL of cell culture corresponding to each BZK concentration, were removed from the 96-well microplates and spotted in TSA 1/10 solid medium, followed by incubation for 24 h at 37°C. The MBC value was defined as the lowest BZK concentration for which bacterial growth was no longer observed.

### Composition of Cellular Aggregates

The polysaccharide (total sugars) content of the aggregate structures formed during long-term incubation of *B. cepacia* and *B. contaminans* isolates in the presence of BZK was assessed using the phenol-sulfuric acid method (Dubois et al., 1956). Bacterial samples were collected from each flask and centrifuged for 5 min at 10,000 rpm, 4°C. The supernatant was discarded, while the pellet was resuspended in 1 mL of dH_2_O. The resulting suspension was mixed with 1.5 mL of phenol 0.05% (w/v) and 8 mL of H_2_SO_4_ 96%. After cooling, the OD_490nm_ was measured. The relative sugar content was determined using a standard curve for the EPS (cepacian) produced by *B. multivorans* (previously *cepacia*) IST408, as previously described (Coutinho et al., 2011a). For protein quantification, the Biuret method was applied (Herbert et al., 1971). Samples were collected from the flasks and centrifuged for 10 min at 4°C, 15,000 rpm. The supernatant was discarded, and the pellet resuspended in 1 mL of dH_2_O. The resultant cell suspension was mixed with 1 mL of dH_2_O and 1 mL of NaOH (1 mM), heated at 100°C for 5 min and let to cool at room temperature. Then, 1 mL of 0.025% CuSO_4_.5H_2_O (w/v) was added, followed by incubation at room temperature for 5 min. After centrifugation at 5,600 g, 20°C, for 5 min, OD_550nm_ was measured. To estimate the protein content, a standard curve for bovine serum albumin (BSA) was prepared.

### Genome Analysis

#### *De novo* Genome Assembly

For genomic DNA extraction, bacterial cultures were prepared by suspending isolated colonies from LB agar plates in 3 mL LB broth, followed by overnight growth at 37°C with shaking at 250 rpm. Genomic DNA was extracted and purified using a DNeasy Blood and Tissue kit (Qiagen, Germany) according to the manufacturer's instructions. DNA concentration and purity were assessed using a Nanodrop ND-1000 spectrophotometer. The draft genome sequences of the *B. cepacia* isolates IST612 and IST701 were obtained using Illumina sequencing technology. An initial quality control of the genome sequences was performed with FastQC (Babraham Bioinformatics). *De novo* assembly of genomes was performed using SPAdes Genome Assembler v3.12.0 (Bankevich et al., 2012). The contigs from the draft genomes were ordered using the MAUVE software (Darling et al., 2004). Further improvement of the assembly was achieved with Pilon (Walker et al., 2014). After the assembly process, a scaffolding step was applied using SSPACE (Boetzer et al., 2010), followed by its improvement with Pilon. Quality control by QUAST (Gurevich et al., 2013) was included after each assembly and scaffolding improvement step.

#### Identification of Genes From the BZK Degradation Pathway

Using NCBI nucleotide BLAST®, the genome sequences of the two *B. cepacia* isolates (IST612 and IST701) were searched for the presence of the 15 genes encoding enzymes reported to be involved in BZK degradation in *B. cenocepacia* AU1054 (Ahn et al., 2016), which was used as a reference strain and its genome retrieved from *Burkholderia* genome database (Winsor et al., 2008). From this analysis, the DNA identity (%) and protein identity and positives (%) of the BZK degradation pathway genes between the reference genome and *B. cepacia* IST isolates was obtained. Artemis Comparison Tool (ACT) (Carver et al., 2005) was used to obtain information regarding putative gene location (in bp), genome inversions and gene order. The BZK degradation enzymes from AU1054 and the IST isolates were compared for their theoretical features using ProtParam tool from ExPASy (Gasteiger et al., 2005) and included as Supplementary Table S1.

## Results

### Long-Term Survival of *B. Cepacia* and *B. Contaminans* Populations During Extended Incubation in Saline Solution Supplemented With BZK

To study the effects of prolonged exposure to stressful environmental conditions, including nutrient starvation and the presence of benzalkonium, mimicking those conditions used during the storage of pharmaceuticals, the *B. cepacia* and *B. contaminans* isolates described in Table 1 were inoculated into glass flasks containing saline solution (NaCl 0.9%). These cell suspensions were either unaltered or supplemented with two levels of BZK [0.0053% (w/v) or 0.05% (w/v)] and kept at 23°C in the dark.

**Figure 1:**
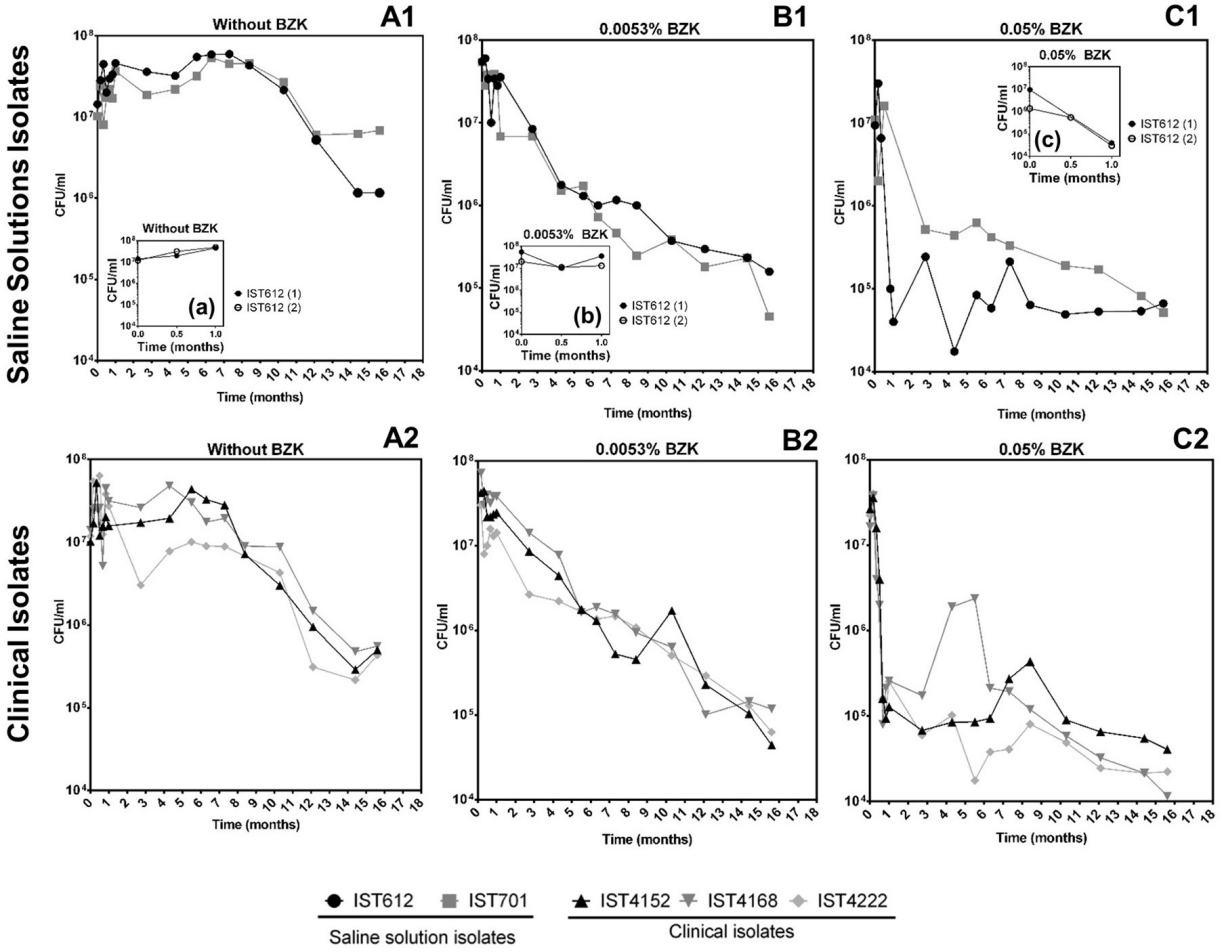
Effect of long-term incubation in saline solutions **(A1,A2)** and saline solutions supplemented with 0.0053% **(B1,B2)** or 0.05% BZK **(C1,C2)** on the viability of five *B. cepacia* isolates, obtained from contaminated batches of saline solutions (IST612 and IST701) and from the sputum of a CF patient (IST4152, IST4168, and IST4222). Cellular viability was assessed as CFUs/mL, obtained through colony counts from three separate plates, corresponding to a range of three different serial dilutions. The inserted panels show results from two independent incubation experiments for *B. cepacia* IST612, during the first month of incubation in each experimental condition (a, b, c).

**Figure 2:**
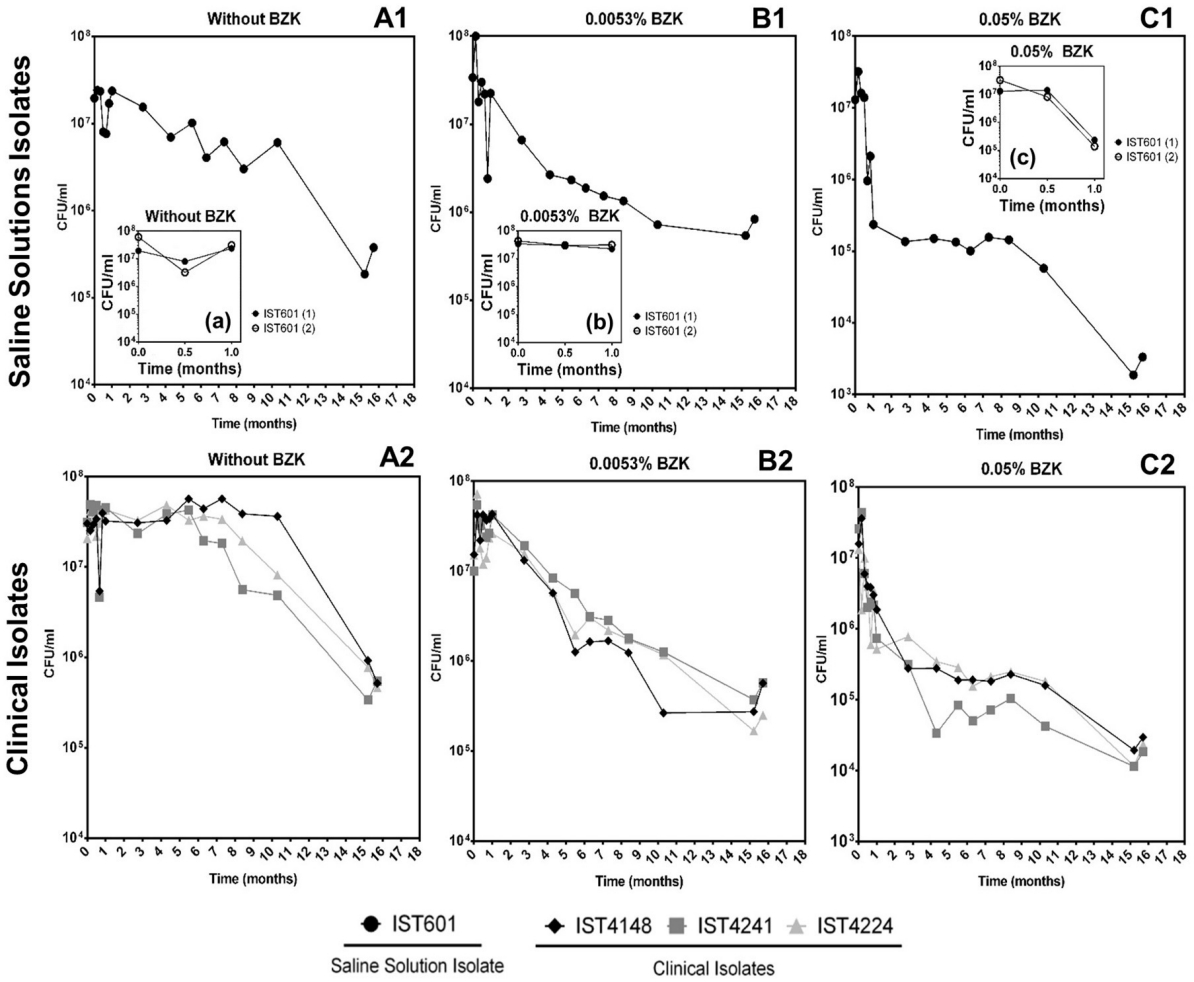
Effect of long-term incubation in saline solutions **(A1,A2)** and saline solutions supplemented with 0.0053% **(B1,B2)** or 0.05% BZK **(C1,C2)** on the viability of four *B. contaminans* isolates, obtained from contaminated batches of saline solutions (IST601) and from the sputum of a CF patient (IST4148, IST4241, and IST4224). Cellular viability was determined as CFUs/mL, obtained through colony counts from three separate plates, corresponding to a range of three different serial dilutions. The inserted panels show results from two independent incubation experiments for *B. contaminans* IST601, during the first month of incubation in each experimental condition (a,b,c).

Cell viability (as colony forming units, CFU/mL) obtained for the 5 clonal variants of *B. cepacia* (Figure 1) and the 4 clonal variants of *B. contaminans* (Figure 2) tested decreased throughout the incubation period (16 months) in a BZK dose-dependent manner. The highest and faster decrease in the viable original population was verified in the presence of BZK (~99.9% for both of the concentrations tested). For 0.05% BZK, this decrease occurred within the first month of incubation, after which the percentage of viable population remained broadly unchanged, corresponding to approximately 0.1% of the initial population. Contrastingly, the viability loss verified for the bacterial populations incubated with 0.0053% BZK was continuous throughout the incubation period and, even after 16 months, the viability of the cellular populations had not yet stabilized.

Bacterial populations incubated in saline solutions (NaCl 0.9%) without BZK had a negligible specific death rates for approximately 10 months, after which a clear and fast decline of the viable bacterial population (in the range of 91–99%) occurred (Figures 1A1,A2, 2A1,A2). The addition of 0.0053% BZK caused a continuous decline of viability during incubation (Figures 1B1,B2, 2B1,B2), and the specific death rate increased at the higher BZK concentration tested. The significant loss of viability was followed by a period of negligible death rate on the addition of 0.05% BZK (Figures 1C1,C2, 2C1,C2) suggesting the development of a persister population.

Differences in the viability decrease profiles obtained for the different clonal isolates (5+4) corresponding to each experimental setting are not considered significant. Although they were single long-term experiments performed at 23°C for more than 1 year, altogether, the results obtained under the different conditions tested validate the average viability profiles (Figure 1C1). Nevertheless, the saline isolates *B. cepacia* IST701 and IST612 exhibited the least similar viability profiles in terms of CFU counts, differing by approximately one order of magnitude for the majority of the timepoints analyzed. Although it is not possible to be conclusive, since we are comparing single experiments, it is interesting to note that the isolate that appears to be more BZK tolerant, IST701, was isolated from a saline solution for nasal application 3 years later than IST612, when the biocide concentration added to the pharmaceutical product was higher. Although other differences could be identified between the two groups of clonal Bcc isolates examined, namely the level of tolerance to the lack of nutrients and presence of different BZK concentrations, the limitations associated with the counting of CFUs/mL draws robust conclusions. In fact, the formation of macroscopically visible cellular aggregates in the presence of BZK (as described below) might lead to an underestimation of cellular viability, and the type of aggregates was found to be species isolate-dependent. Despite thorough homogenization by vortexing the diluted samples prior to plating, in order to disrupt the aggregate structures, heterogeneity concerning the number/size of the aggregates in each sample is anticipated. This limitation contributes to the variation among experimental CFU values, especially registered for samples obtained from saline solutions supplemented with higher BZK concentrations after extended incubation periods, where the number and size of the cell flocks increase.

The minimum inhibitory concentrations (MIC) and minimum bactericidal concentrations (MBC) were determined for several isolates, also mimicking the storage conditions for saline solutions (23°C) (Table 2). However, these values were obtained in growth medium, while the long-term viability experiments were performed under nutrient limitation in a saline solution. The MIC values obtained are situated within the upper range of values previously reported in the literature for *B. cepacia* (50–200 μg/mL) (Rose et al., 2009; Kim et al., 2015). Differences between the clonal isolates of the two Bcc species tested were also detected, with the *B. cepacia* isolates displaying higher resistance to BZK in comparison with the *B. contaminans* isolates tested.

**Table 2:** MIC and MBC values toward BZK obtained for *B. cepacia* and *B. contaminans* isolates examined in this study.

**Strain**	**Original isolate**	**MIC (μg/mL)**	**MBC (μg/mL)**
*B. cepacia*	IST612	192	192
	IST4152	192	192
	IST4168	192	192
*B. contaminans*	IST601	128	128
	IST4241	128	128
	IST4224	128	128

*Values are means of at least two replicates and three biologically independent experiments*.

The MBC and MIC values were the same for all the isolates analyzed, consistent with what has been reported for other relevant Gram-negative microbial pathogens when exposed to BZK (Morrissey et al., 2014), since for bactericidal drugs like BZK, the MBC values are usually the same as the MIC values, differing by no more than four times the MIC.

### Colony Size Reduction and Other Morphology Alterations Observed During Extended Incubation in Saline Solutions Supplemented With BZK

**Figure 3:**
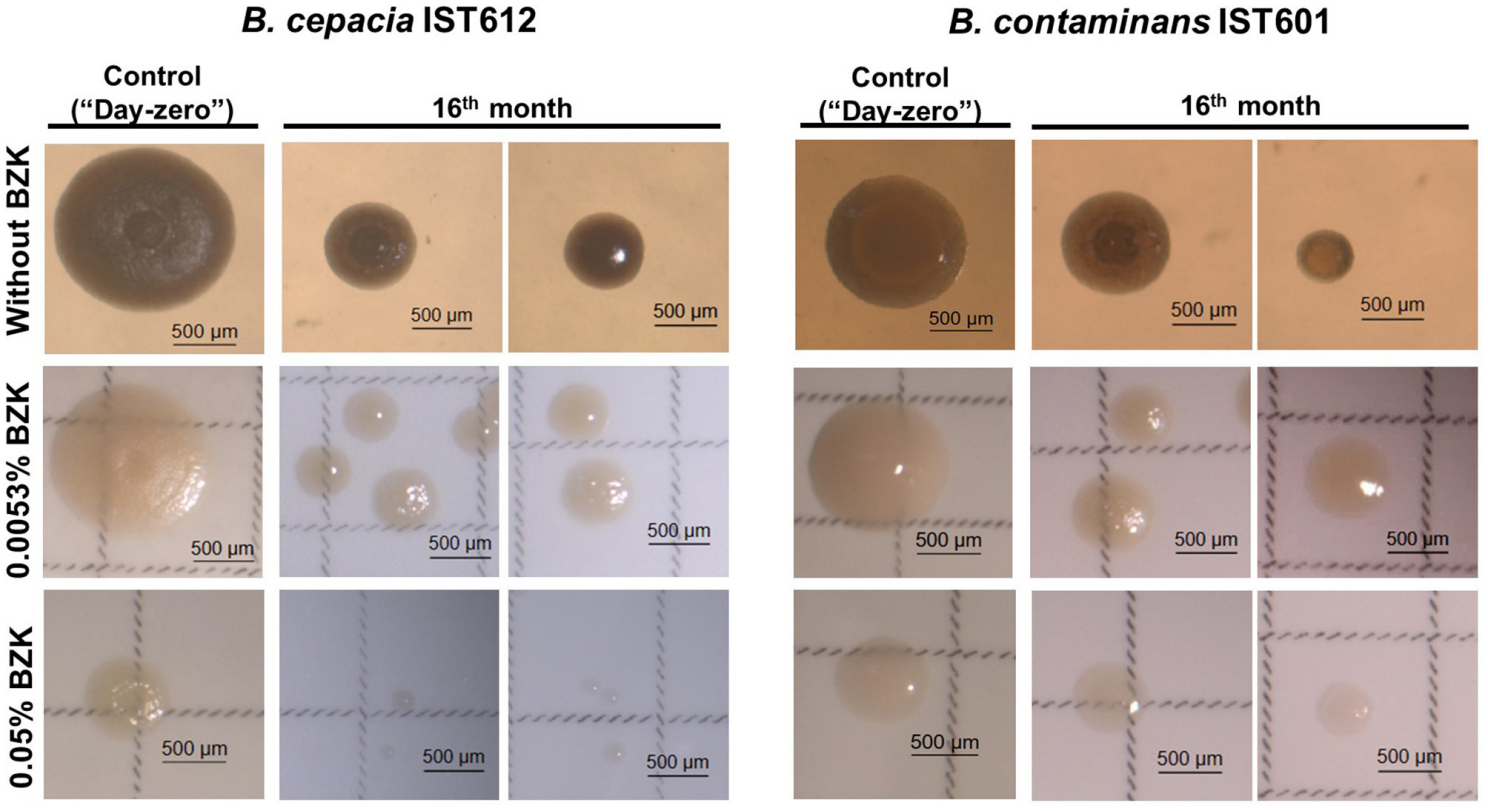
Different colony morphotypes exhibited by *B. cepacia* IST612 and *B. contaminans* IST601 cellular populations incubated in saline solutions without BZK, and in saline solutions supplemented with 0.0053% or 0.05% BZK, after 16 months of incubation. Colony morphologies were compared with those obtained for the same bacterial isolates at initial inoculation (“day-zero”) in the same conditions. It is possible to observe differences in the colony size, morphology, and pigmentation.

Different incubation conditions resulted in the development of heterogeneous bacterial populations, characterized by major phenotypic differences, including a diversification of colony morphotypes and a clear decrease in colony diameter. At initial inoculation (“Day-zero”), all of the colonies exhibited a similar rough morphology and size (Figure 3). After 16 months of incubation in saline solutions without BZK, most colonies remained rough (~95.5%), but some smooth colonies were observed (~4.5%). More smooth colonies evolved in 0.0053% BZK (~68.7%), while the higher concentration of BZK (0.05%) led to complete replacement of the rough morphology with smooth colonies (Figure 3). Evolved colonies also lost pigmentation and, in the case of *B. contaminans* isolates, became more mucoid than *B. cepacia* isolates (Figure 3).

**Figure 4:**
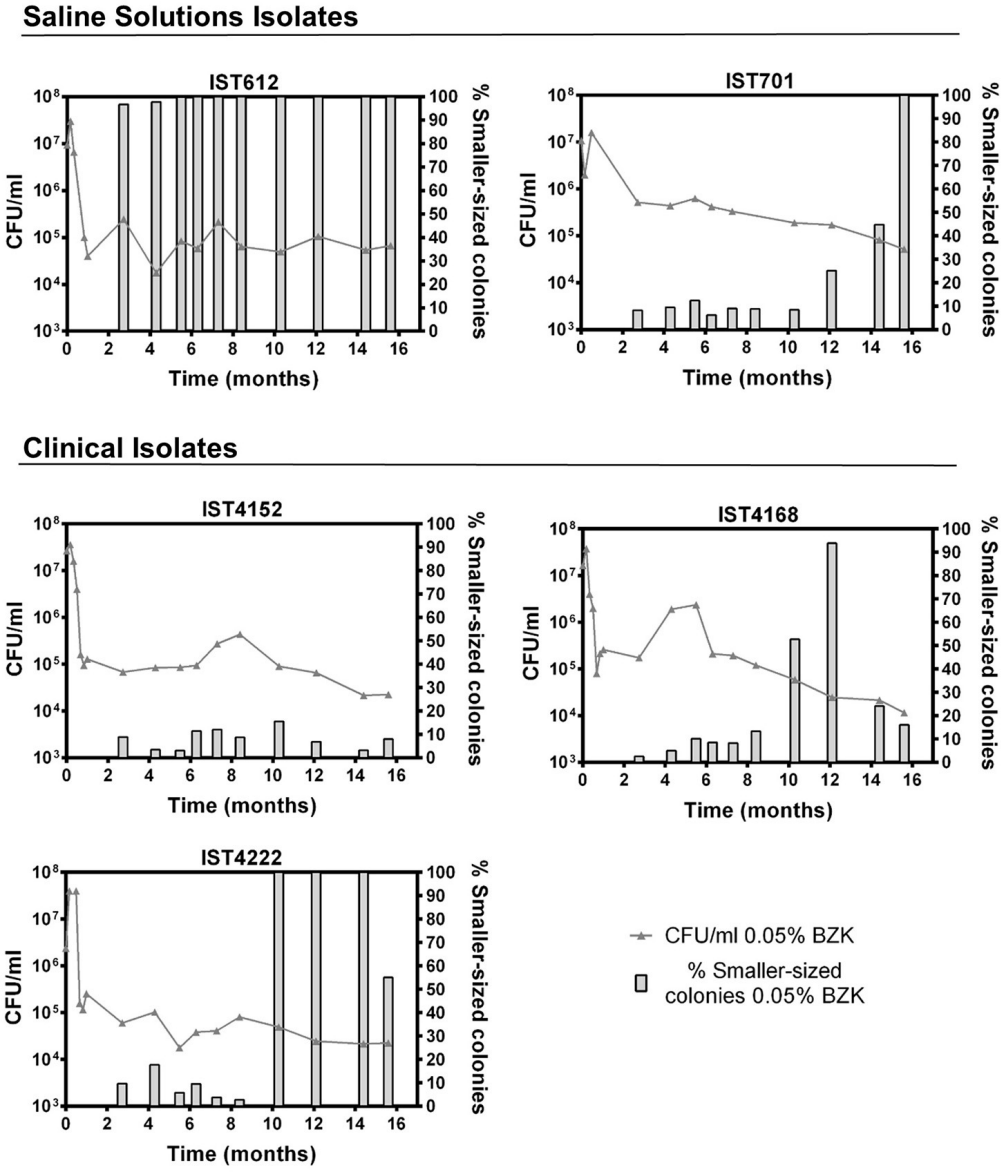
Evolution of cell viability (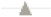
0.05% BZK) and percentage of smaller-sized colonies (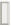
% Smaller-sized colonies 0.05% BZK) obtained for the *B. cepacia* bacterial populations during 16 months of incubation in saline solutions containing 0.05% BZK. The percentage of smaller-sized colonies only started to be registered at the 82nd day. Cell viability values hereby plotted correspond to those represented in Figure 1.

**Figure 5:**
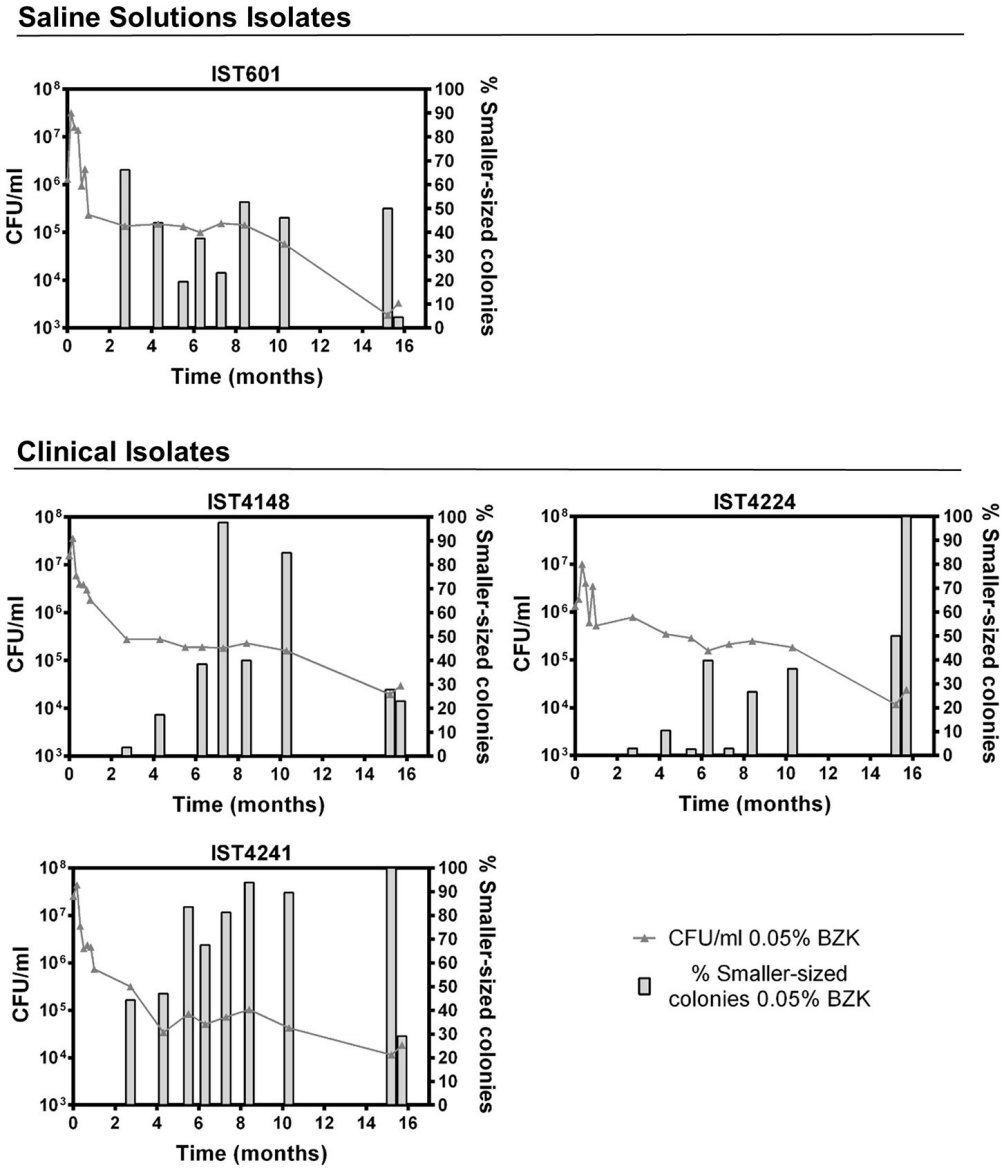
Evolution of cell viability (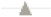 0.05% BZK) and percentage of smaller-sized colonies (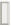 % Smaller-sized colonies 0.05% BZK) obtained for the *B. contaminans* bacterial populations during 16 months of incubation in saline solutions containing 0.05% BZK. The percentage of smaller-sized colonies only started to be registered at the 82nd day. Cell viability values hereby plotted correspond to those represented in Figure 2.

**Figure 6:**
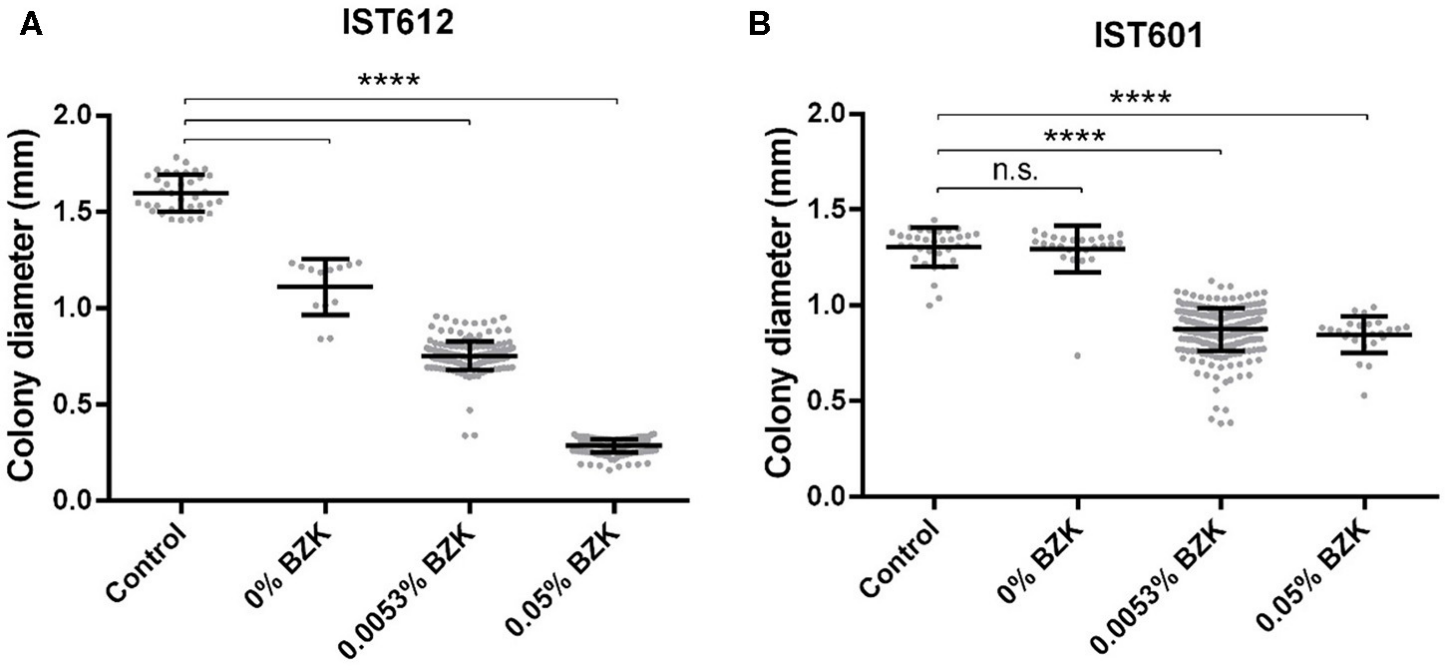
Colony diameter distribution of the cell populations corresponding to *B. cepacia* IST612 **(A)** and *B. contaminans* IST601 **(B)** incubated for 16 months under three different stress conditions. The colony diameters of the original isolates, grown on LB medium, were also assessed and used as a control. From 30 to 260 individual colonies were selected for measurement. Mean colony diameters ± SD are also plotted. The results of the Mann–Whitney *U*-test (****P < 0.0001, n.s. not significant) are indicated.

Long-term incubation in the aforementioned conditions also led to colony size variation, as evidenced by the emergence of smaller-sized colonies within the first 30 days of the experiment. However, after the 82nd day of incubation, their percentage increased significantly and was quantified (Figures 4, 5). Although small colonies (≤ 0.5 mm in diameter) were also observed in the absence of BZK, they were much more common in the presence of the preservative, representing between 5 and 100% of the total number of colonies, depending on the Bcc isolate tested (Figures 4, 5). After 48 h of incubation, colonies obtained from saline solutions exhibited an average diameter of 1.6 mm (±0.03), while colonies isolated from solutions containing 0.0053 and 0.05% BZK displayed mean diameters of 1.5 mm (±0.02) and 1.1 mm (±0.06), respectively. The development of smaller-sized colonies, as a result of the extended incubation period, translated into a decrease of colony size of approximately 26%, in the case of bacterial populations incubated in saline solutions without BZK. This decrease was more significant for cellular populations incubated with 0.0053 and 0.05% BZK, corresponding to ~45 and ~52%, respectively. The overall decrease in colony size was a dose-dependent response to higher BZK concentrations for both *B. cepacia* and *B. contaminans* isolates (Figure 6). However, cell populations of *B. contaminans* incubated in the presence of the same BZK concentration were generally composed by bigger colonies, when compared to the *B. cepacia* populations (Figure 6). A correlation between the frequency of appearance of small-sized colonies and the percentage of the persistent population could not be established.

Smaller-sized colonies isolated from saline solutions without BZK were found to revert to the original phenotype after the 2^nd^ passage in a stress-free environment (TSA plates). The same was generally observed for smaller colonies isolated from saline solution containing 0.0053% BZK. However, colonies obtained from saline solutions supplemented with the highest biocide concentration (0.05%) maintained the smaller phenotype, which indicates that, in this particular condition, the smaller colony size might become an inheritable trait. The results indicate that the stability of the smaller colony size phenotype is influenced by the intensity of the stress condition to which cells are exposed. It should also be noted that colonies exhibiting diameters ≤ 0.5 mm were considered as smaller-sized colonies, while only those presenting one-tenth the size of the original colonies were termed small colony variants (SCVs), by definition (Proctor et al., 2006).

### Reduction of Cell Size During Extended Incubation in Saline Solution Supplemented With BZK

**Figure 7:**
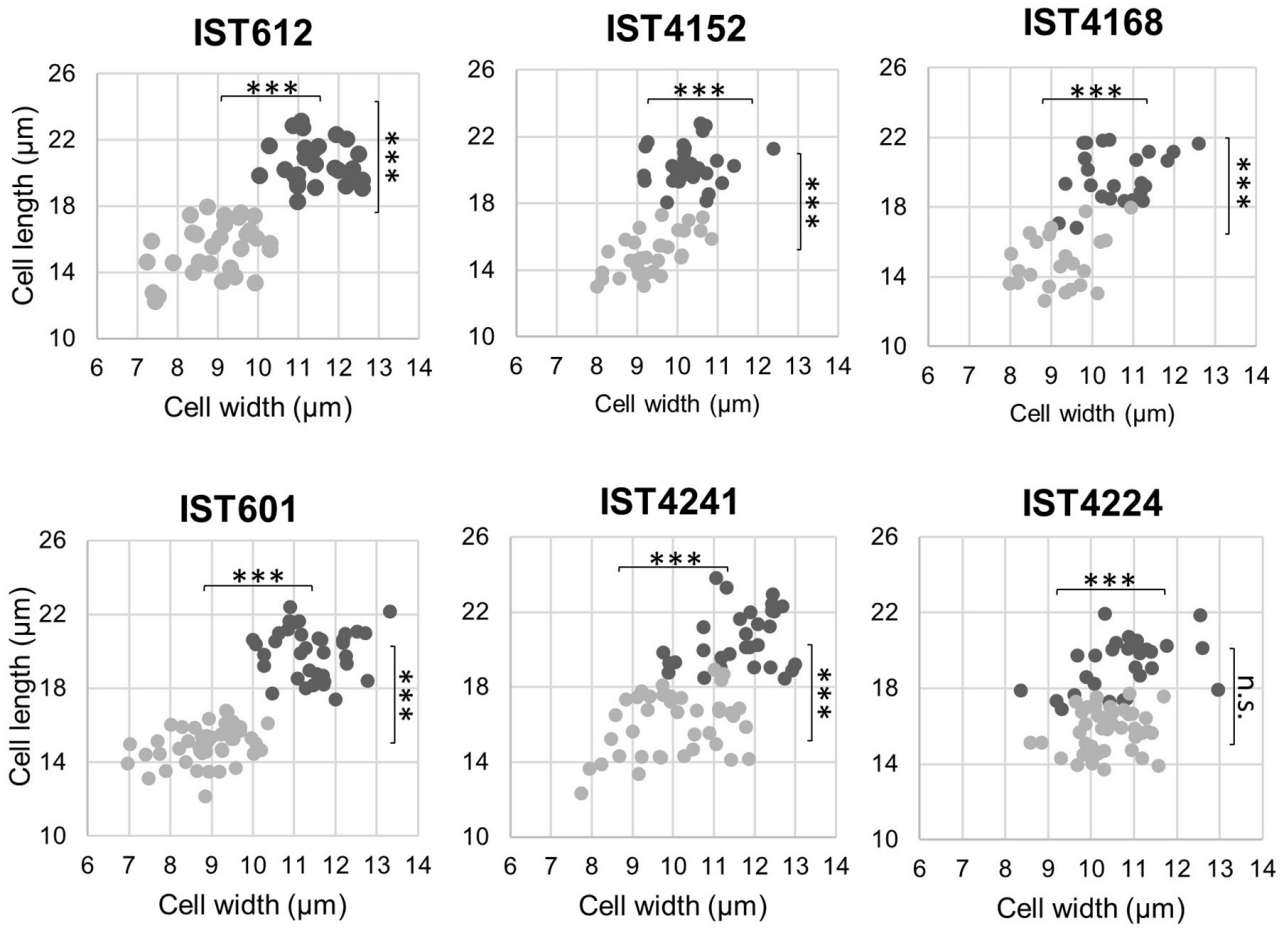
Scatter plot of cell length and width of *B. cepacia* (IST612, IST4152, and IST4168) and *B. contaminans* (IST601, IST4241, and IST4224) populations recovered from saline solutions containing 0.05% BZK after 3 months of incubation, corresponding to the normal sized clonal variant (dark gray) and to the small clonal variant (light gray). The results of the Mann-Whitney u-test (***P ≤ 0.001, n.s. not significant) are indicated.

Besides the observed effects exerted by extended incubation in saline solutions supplemented with 0.05% BZK on the reduction of colony size, the presence of BZK also induced a decrease of cell size, as observed using fluorescence microscopy. Cell length and width measurements indicated that unlike normal-sized colonies, smaller-sized colonies of both *B. cepacia* and *B. contaminans* were composed of smaller cells with an atypical shape, closer to coccus/coccobacilli (Figure 7). After 3 months of incubation, the average surface area and volume of normal cells were 546.1 (±51.2) μm^2^ and 2945.6 (± 412.1) μm^3^, respectively, while the average surface area of smaller cells was 348.8 (± 55.0) μm^2^ and the average volume was 1360.3 (± 348.7) μm^3^ (Figure 7). Thus, surface area is reduced, on average, by ~35% and volume by ~55%.

### BZK as an Inducer of the Formation of *B. cepacia* and *B. contaminans* Aggregates and Their Characterization

Throughout the course of the incubation experiments, it was possible to observe, with the naked eye, the formation of a white material that deposited on the bottom of the flasks containing 0.05% BZK, after the 30th day of incubation.

**Figure 8:**
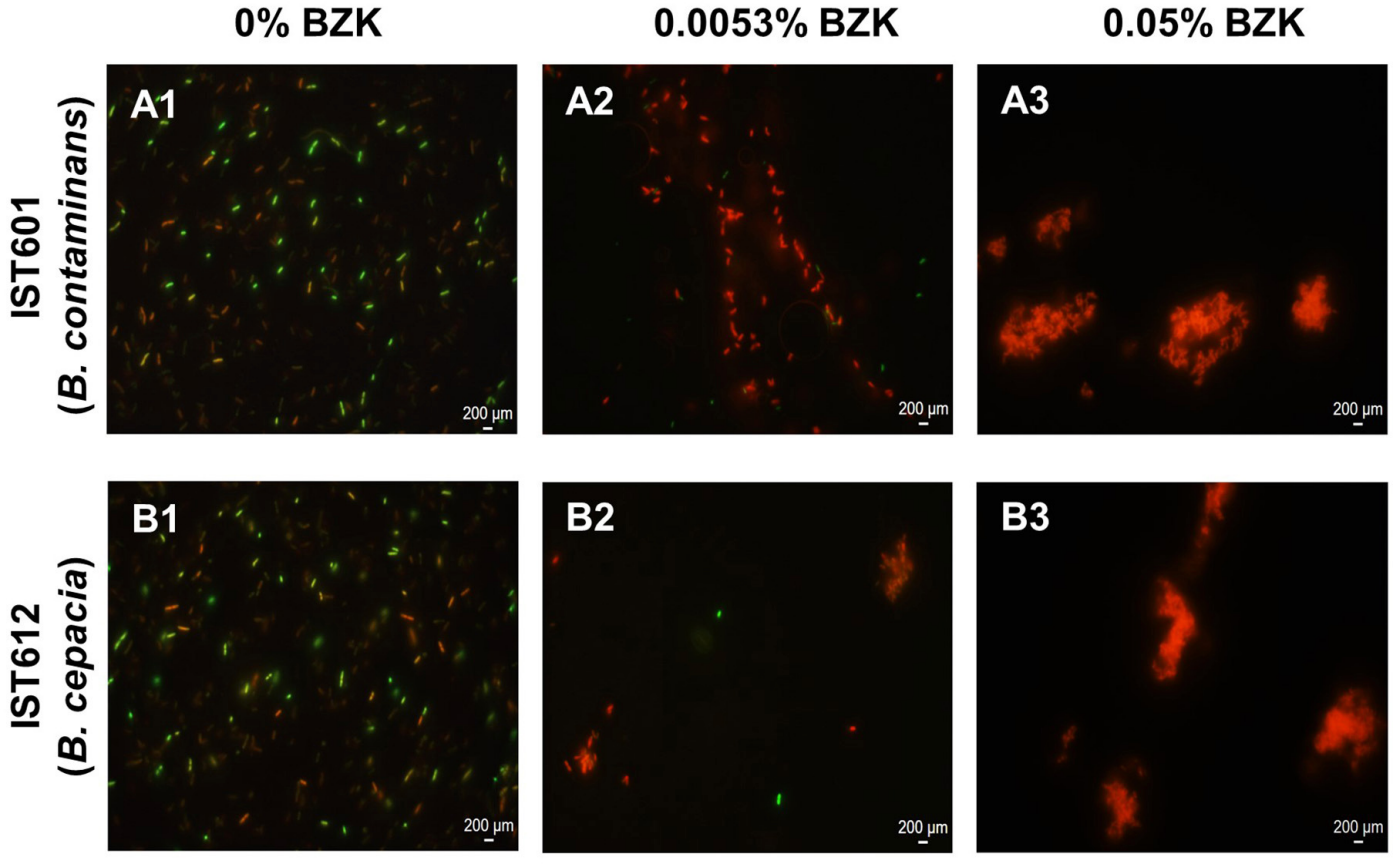
Microscopic observation of the structures formed by *B. cepacia* IST612 and *B. contaminans* IST601, after 16 months of incubation in saline solutions supplemented with increasing concentrations of BZK. **(A1,B1)** Control cells, incubated in saline solution (NaCl 0.9% (w/v)); **(A2,B2)** Cells incubated in saline solution supplemented with 0.0053% (w/v) of BZK; **(A3,B3)** Cells incubated in saline solution supplemented with 0.05% (w/v) of BZK. Cellular viability was assessed through co-staining of cells with SYTO 9 (green) and propidium iodide (red). Images were acquired using a fluorescence microscope (the same settings were applied for each image) and are equally scaled to allow direct comparison.

Microscopic observation of samples from cell suspensions belonging to IST612 (*B. cepacia*) and IST601 (*B. contaminans*) was performed using fluorescence microscopy, after 16 months of incubation in the three stress conditions described, where live cells were stained with SYTO 9 (green) and dead cells were stained with propidium iodide (red) (Figure 8). Isolates incubated in saline solutions without BZK exhibit a dispersed cellular population, essentially composed of single cells (Figures 8A1,B1). *B. cepacia* bacterial populations, incubated in the presence of 0.0053% BZK, were generally composed by cellular aggregates of intermediate size, with mostly dead or membrane compromised cells. Nevertheless, some live cells were spotted, residing within those aggregates (Figure 8B2). The *B. contaminans* population that was incubated in the same conditions did not display any aggregates of considerable size and were composed of small groups of 2–5 cells (Figure 8A2). Most cells were also dead/membrane compromised. For the highest BZK concentration (0.05%), the bacterial populations showed a clear tendency to form denser and bigger aggregate structures (Figures 8A3,B3). In general, the *B. cepacia* isolates incubated in 0.05% BZK exhibited a homogeneously turbid appearance, while the *B. contaminans* isolates formed visible floccular structures.

**Figure 9:**
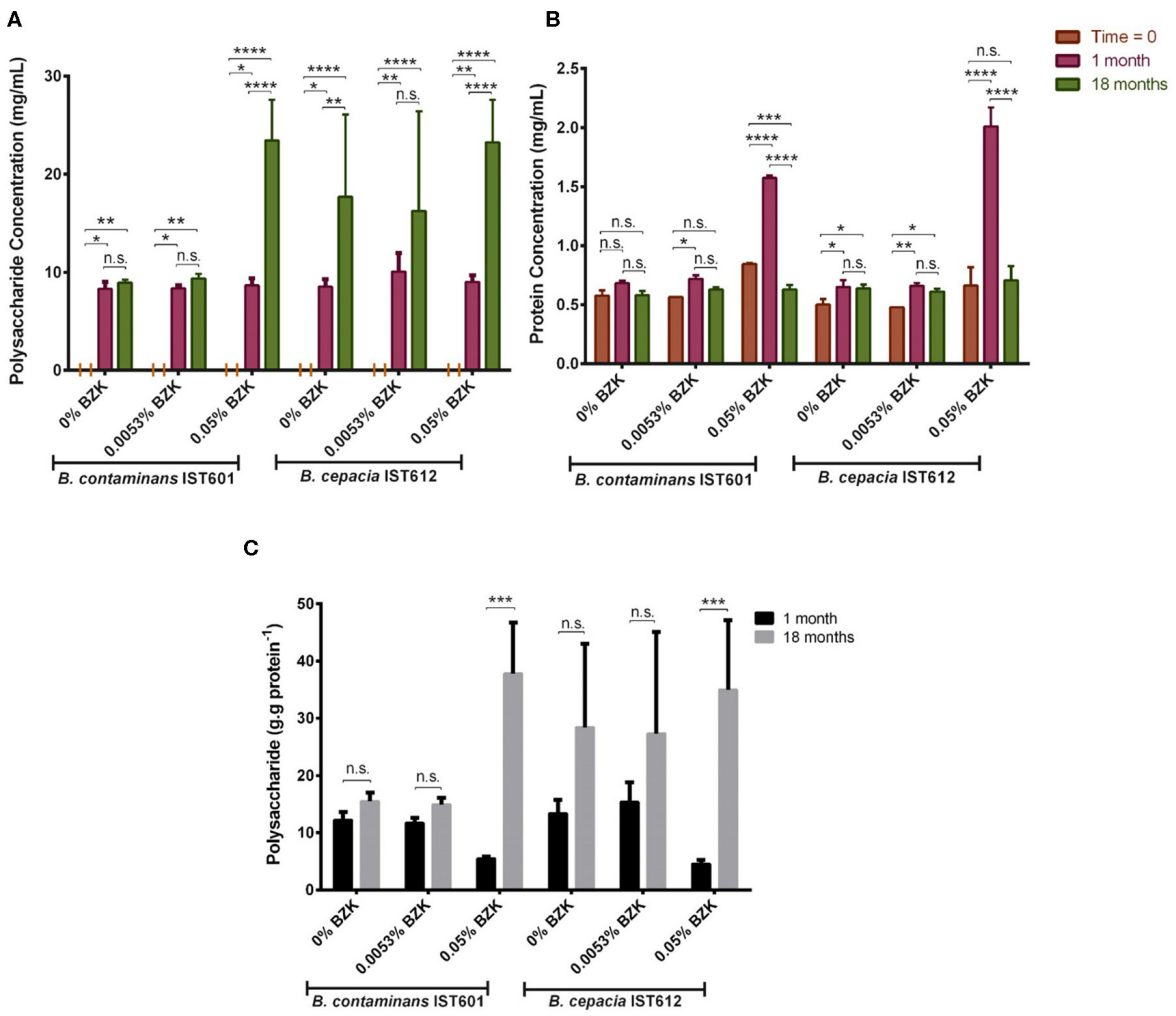
Levels of polysaccharide **(A)**, protein **(B)** and the ratio of polysaccharide vs. protein **(C)** produced by *B. cepacia* IST612 and the *B. contaminans* IST601, assessed by the phenol-sulphuric and biuret methods, respectively, and expressed in mg per mL of bacterial sample, at initial incubation (“Time = 0”) and after, 1 and 18 months of incubation in saline solutions and saline solutions supplemented with 0.0053% (w/v) or 0.05% (w/v) BZK. The results are means of two independent experiments with three replicates each. At initial inoculation (“Time = 0”) no polysaccharide content was detected. The results of the 2-way ANOVA test (*P < 0.05, **P ≤ 0.01, ***P ≤ 0.001, ****P < 0.0001, ns not significant) are indicated.

To assess the composition of the aggregate-like structures produced by the Bcc isolates under study, triggered by BZK, the ratio between polysaccharide and protein concentration was determined (Figure 9). After 18 months of incubation in saline solutions or saline solutions containing two different BZK concentrations, the polysaccharide content of the cellular aggregates was higher, in comparison with the values obtained after one month, for both *B. cepacia* and *B. contaminans* populations (Figure 9A). At initial inoculation (“time zero”), polysaccharide was not detected within the bacterial samples analyzed (Figure 9A). The results indicate that the composition of the cellular aggregate's matrix changed over time, becoming richer in (poly)saccharide during long-term incubation, as indicated by the polysaccharide vs. protein plot (Figure 9C). This increase was particularly notorious for the highest 0.05% BZK, for which the polysaccharide content increased the most between the three time-points analyzed (Figure 9A). These observations suggest that cellular populations adapt to the presence of higher BZK concentrations by producing aggregates rich in polysaccharide, which is also corroborated by the macroscopic observation of these structures. The cellular protein concentration remained relatively constant throughout the incubation time, for both cellular populations incubated either without BZK or with the lower concentration (0.0053% w/v) of the biocide (Figure 9B). The increase in the protein concentration registered after 1 month of incubation with 0.05% BZK suggest that BZK may be used as carbon and energy source by Bcc bacteria as reported before (Geftic et al., 1979). The decrease of protein concentration registered at the 18th month of incubation with 0.05% BZK could be due to protein degradation (Figure 9B).

### Identification of a Putative BZK Degradation Pathway in *B. cepacia* Isolates

**Figure 10:**
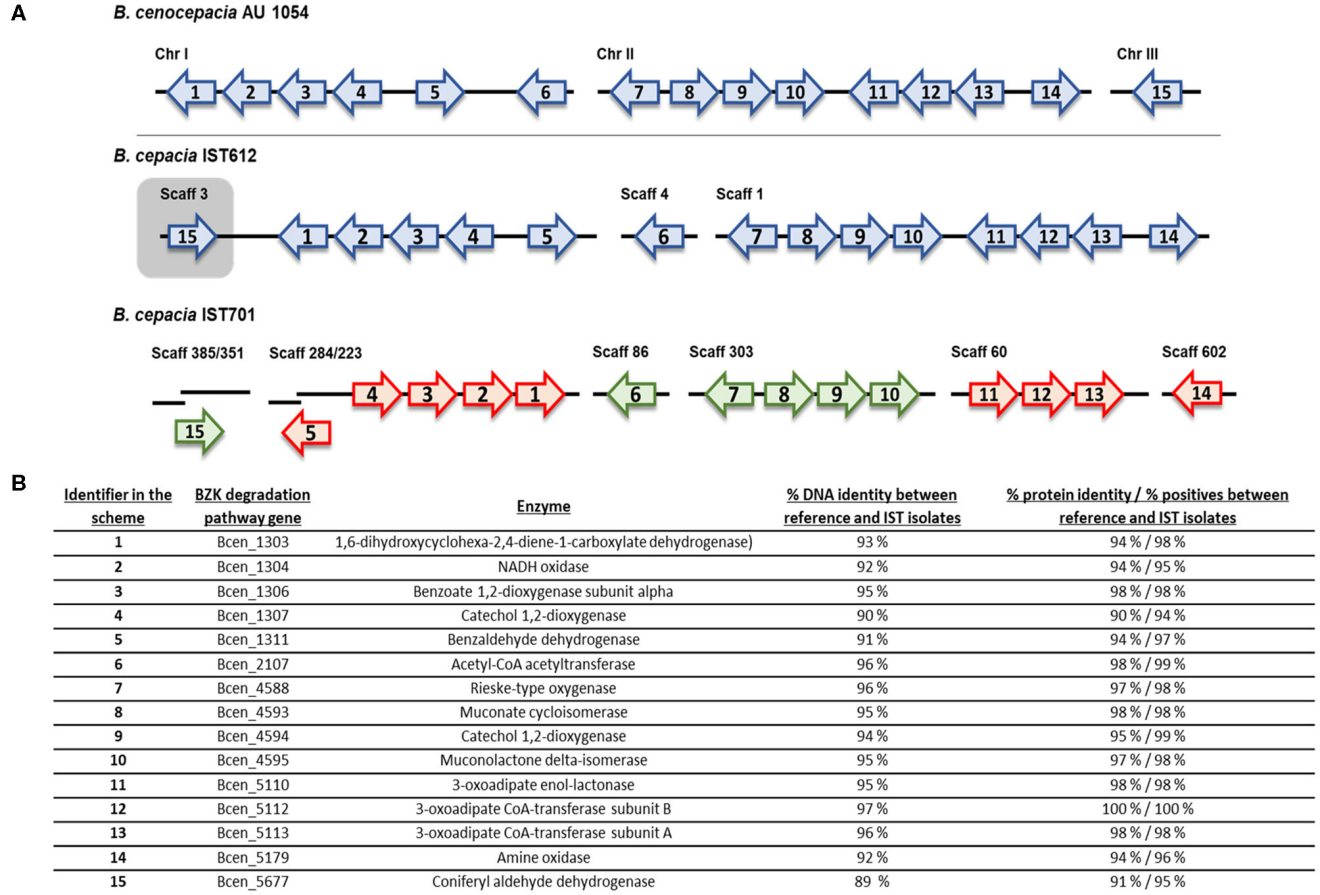
Genomic organization and transcriptional orientation of the 15 homologs of the *B. cenocepacia* AU1054 BZK catabolism genes, identified within the genomes of *B. cepacia* IST612 and IST701. Genes are represented by labeled arrows **(A)** and their respective locus-tag, products, percentage DNA identity, and percentage protein identity and positives (from BLASTp tool) between reference and IST isolates are listed below **(B)**. The IST701 BZK catabolism genes are colored in green and red, depending on whether the genes are encoded in the same or reversed transcriptional orientation of that of IST612, respectively. Gene size, distance and positioning in the chromosomes and scaffolds are not to scale.

To confirm the existence of a metabolic pathway responsible for BZK degradation, two *B. cepacia* isolates, IST612 and IST701, recovered from intrinsically contaminated saline solutions in 2003 and 2006, respectively, were selected for genomic analysis. The genome sequences of those isolates were compared against the nucleotide sequences of 15 genes described to be involved in BZK degradation in *B. cenocepacia* AU1054 (Ahn et al., 2016), using the NCBI nucleotide BLAST tool. All the 15 genes encoding BZK degradative enzymes have corresponding homologs in the *B. cepacia* IST612 and IST701 genomes (Figure 10), confirming the existence of a degradation pathway for BZK.

Further characterization of the BZK degradation pathway genes aimed at inferring gene organization and synteny, and to assess the occurrence of genomic rearrangements. The BZK degradation pathway genes were found to be distributed across the three chromosomes of *B. cenocepacia* AU1054, with a given transcriptional orientation (Figure 10A). In the draft genome sequence of *B. cepacia* IST612, we were able to find the homolog genes for the BZK degradation pathways distributed across scaffolds 1, 3, and 4, with 14 of the genes showing the same transcriptional orientation as in the AU1054 genome sequence. The homolog gene to Bcen_5677 (identified in the scheme as 15), encoding an aldehyde dehydrogenase, was found in the same scaffold as the genes of chromosome I of AU1054, with a reversed transcriptional orientation when comparing to AU1054. Considering that in the genome of AU1054, Bcen_5677 is found in chromosome III, and that IST612 sequence is an Illumina draft genome, this reversed transcriptional orientation may be an artifact from the scaffold definition process. In the draft genome sequence of *B. cepacia* IST701, the BZK degradation genes are distributed across 8 scaffolds, with two of the genes divided in two scaffolds. Comparing with the transcriptional orientation of the genes in the IST612 genome, there are 9 genes with reversed transcriptional orientation in the IST701 genome, strongly indicating several genome rearrangements of large genome sequences (Figure 10A). Although no SNPs were found among the two IST isolates for the BZK degradation pathway genes, these isolates' genes share DNA percentage identities varying between 89 and 96 and protein percentage identity varying between 90 and 100% identity with *B. cenocepacia* AU1054 gene sequences (Figure 10B). The positive percentage provided by NCBI BLASTp® tool, that accounts for the type of amino acid substitution, indicate protein similarities equal or higher than 94%, emphasizing the high conservation of the protein sequences, as also documented by the protein theoretical features (Supplementary Table S1).

## Discussion

Bacteria of the *Burkholderia cepacia* complex (Bcc) are characterized by a remarkable metabolic diversity and persistence and survival in nutrient-limited aqueous environments. For that reason, water raises a particular concern in industrial pharmaceutical settings, where it is used as a raw material (Torbeck et al., 2011; Cundell, 2019; Tavares et al., 2020). In fact, pharmaceutical-grade water has been identified as the underlying cause behind several Bcc outbreaks related to the use of contaminated products (Torbeck et al., 2011; Cundell, 2019). Moreover, the lack of good manufacturing practices in industrial settings, including deficient quality control and poorly designed water systems, are also among the reasons behind the presence of Bcc in pharmaceutical products (Ali, 2016). The present work provides insights into the phenotypic changes that occurred during the adaptation of different clonal isolates of *B. cepacia* and *B. contaminans* to long-term incubation in saline solutions and in saline solutions supplemented with different concentrations of benzalkonium chloride (BZK), mimicking the effects of nutrient starvation and the presence of this biocide in pharmaceutical product formulations.

The isolates examined in this work are epidemiologically related and were obtained from batches of contaminated saline solutions for nasal application or from the sputa of two CF patients (Cunha et al., 2003, 2007; Coutinho et al., 2015). Despite the 16 months of incubation in saline solution without any source of carbon or energy, under the test conditions, we found that viable cells of *B. cepacia* and *B. contaminans* could still persist and be recovered using a culture-based method. The same was observed for isolates maintained in saline solutions containing 0.0053% or 0.05% BZK. For the higher BZK concentration, a period of rapid exponential death with a duration of approximately 1 month was followed by a stabilization of the CFU counts. This biphasic killing curve is characteristic of the enrichment of the original population with persister cells (Fauvart et al., 2011; Maisonneuve and Gerdes, 2014; van den Bergh et al., 2017) and was accompanied by marked phenotypic alterations of colony and cell morphology and aggregation, as discussed later. Previous reports have also shown that Bcc species can survive and remain viable for prolonged periods of time in water without any source of nutrients (Carson et al., 1973; Zanetti et al., 2000; Vermis et al., 2003; Moore et al., 2008; Pumpuang et al., 2011; Torbeck et al., 2011; Gilbert and Rose, 2012; Ahn et al., 2014) and a “*Pseudomonas cepacia”* strain survived during 14 years of incubation in an ammonium acetate solution supplemented with 0.05% BZK (Geftic et al., 1979). Since BZK is used in a variety of commercial products in concentrations ranging from 0.02 to 5% (200–50,000 μg/mL) (Kim et al., 2015) and as a preservative in concentrations between 0.004 and 0.02% (40–200 μg/mL) (Kaur et al., 2009), the isolates studied in this work would have not been killed at the lowest commercially used concentrations or at any preservative concentrations.

Long-term incubation in the different stress conditions examined in this study resulted in the appearance of distinct colony morphotypes. Colonies isolated from saline solutions under nutrient starvation produced a rough and nonmucoid morphology throughout the experiment. However, incubation in the presence of BZK was associated with a dose-dependent smoother and mucoid phenotypes. In Bcc bacteria, the transition from a mucoid to a nonmucoid phenotype is more common in the context of CF infections, where the nonmucoid phenotype is associated with increased virulence, leading to a faster lung function decline (Zlosnik et al., 2008, 2011). In contrast, the mucoid phenotype is associated with persistence and decreased virulence, which might be more favorable in the presence of BZK, that can be used as source of carbon and energy for exopolysaccharide biosynthesis, as our results suggest.

A clear tendency for a gradual and consistent decrease in colony size during long-term incubation in saline solutions was also observed. This alteration pattern was further enhanced in the presence of BZK in a dose-dependent manner. However, the development of colony variants that can really be considered as small colony variants (SCVs), consisting of slow-growing bacterial subpopulations characterized by being one-tenth the size of the original-type bacteria (Proctor et al., 2006; Johns et al., 2015), was only observed to arise for the higher BZK concentrations (0.05%), constituting 100% of *B. cepacia* isolates IST612, IST701 and *B. contaminans* isolate IST4241 populations, after 16 months of incubation. Remarkably, the smaller-sized variants isolated from saline solutions without BZK or containing 0.0053% BZK (26–45% size reduction in comparison to the original colonies), reverted to the original morphology immediately after the second passage in optimal growth conditions. It is likely that the SCVs that emerged upon adaptation to higher BZK concentrations have undergone more permanent genetic changes, maintaining the SCV phenotype. SCVs usually have deficiencies in the electron transport chain, which translate into lower ATP production and reduced metabolism (Proctor et al., 2006; Johns et al., 2015). Since ATP production is essential for cell wall and membrane biosynthesis, as well as for pigment production, the “dormancy” state that characterizes SCVs might explain the reduced size of the colonies formed and specific growth rate, and the lack of pigmentation (Proctor et al., 2006; Johns et al., 2015). Conversion into a state of low metabolic activity (dormancy) contributes to bacterial persistence and tolerance to antimicrobial agents (Maisonneuve and Gerdes, 2014; Fisher et al., 2017), leading to recurrent and often fatal infections (Häussler et al., 2003; Malone, 2015).

In terms of length and width, cells also became more rounded/coccoid in response to BZK. Many Gram-negative pathogens develop smaller coccoid forms in response to stress, such as long-term incubation in fresh or salt water (Yang et al., 2016). Since nutrient acquisition relies on diffusion and capture of molecules at the cell surface, smaller cells can capture molecules present in very low concentrations to support their metabolic requirements (Bergkessel et al., 2016). The relationship between cell size and nutrient availability may be related with fatty acid biosynthesis. A model proposed for *Escherichia coli* suggests that the flux of nutrients into the cell controls the synthesis of fatty acids which, in turn, controls cell size through modeling of the cell membrane surface area and cytoplasmic volume (Yao et al., 2012). A recent study performed in our lab also demonstrated a clear length and height decrease of *B. cenocepacia* cells during more than 3 years of chronic infection, suggesting that the population converged evolutionarily toward the minimization of bacterial size (Hassan et al., 2019). Small microbial cell size might allow evasion from important host defenses and cell shape itself has been suggested as a virulence trait (Vadia et al., 2017). Therefore, the progressive decrease of cell size induced by BZK might favor persistent infection and more efficient nutrient acquisition, suggesting an additional danger if contaminated saline solutions are used by CF patients.

One of the most striking observations from this work was the formation of macroscopically visible cellular aggregate-like structures, in the presence of the higher biocide concentration tested (0.05% w/v), especially for the *B. contaminans* isolates' populations. The characterization of the aggregates' matrix revealed that these structures were rich in (poly)saccharide. Moreover, the polysaccharide content significantly increased during long-term incubation, a tendency that was accompanied by the development of visually denser cellular aggregates. This biomass increase suggests that BZK might be catabolized and used as carbon and energy source. Since polysaccharide and cell aggregates' production implies a significant carbon and energy expense, the low polysaccharide levels displayed by the bacterial populations incubated in the absence of BZK or with the lower BZK concentration is consistent with the carbon and energy starvation/limitation expected in these environments. Overall, in the presence of BZK, polysaccharide production might constitute a long-term adaptation mechanism, allowing the maturation of the aggregates' structure and increasing the chances of bacterial survival. Although long-term exposure to BZK chloride was previously associated with an evolutionary response of a microbial community toward multidrug resistance (Tandukar et al., 2013), in the present study it is for the first time described the impact that the presence of BKZ has in Bcc aggregate formation. In saline incubation media with no source of carbon and energy, *B. cepacia* and *B. contaminans* bacterial populations were also able to survive, implying a “sacrifice-for-survival” mechanism (Snoussi et al., 2018), in which the intracellular components released by dead cells might be used to maintain a smaller viable population. Additionally, and despite the dose-dependent BZK's deleterious effects that stimulate an initial viability loss, after adaptation to the biocide, especially at the maximum concentration, it was likely used as carbon and energy source to maintain cellular viability and support polysaccharide-rich cellular aggregates' formation during extended incubation. These results strongly suggest that BZK can be catabolized by *B. cepacia* and *B. contaminans*, even when present as the sole carbon and energy source. Biodegradation of Benzalkonium chlorides has also been reported to occur within microbial communities containing members of the *Burkholderia* genus (Oh et al., 2014). Those microbial communities were able to metabolize Benzalkonium chlorides as a sole carbon and energy source through dealkylation reactions, within 12 h (Oh et al., 2014). Metatranscriptomic studies revealed that the dealkylation process was mediated by an amine oxidase, while several other enzymes involved in energy generation processes (such as the TCA cycle) and fatty acid metabolism, as well as monooxygenases, dioxygenases, and dehydrogenases were also upregulated upon BAC exposure (Oh et al., 2014). When in microbial communities or as isolated cells, Bcc strains were found to possess high inherent tolerance to BZK, a feature attributed to the activity of efflux pumps and their capacity to catabolize BZK (Ahn et al., 2016). In fact, the N dealkylation-dependent degradation of BZK by *B. cenocepacia* was identified as an effective detoxification process and metabolic benefit for the use of an alternative energy and carbon source, and the 15 genes involved in the degradation pathways were identified in strain AU1054 genome (Ahn et al., 2016). In the present study, these 15 genes were found to be present in the genomes of the *B. cepacia* IST isolates tested, as suggested by the high percentage of DNA and protein identity with AU1054 catabolic genes/proteins. In summary, the BZK-dose dependent increase of biomass and polysaccharide-rich aggregates after extended incubation in saline solution with this biocide together with the presence in the genome of the *B cepacia* IST isolates of genes/proteins with a very high DNA/protein sequence identity to those of the BZK degradation pathway previously identified in *B. cenocepacia* AU 1054, strongly support our hypothesis that BZK is used as carbon- and energy- source producing those polysaccharide-rich-cell aggregates.

This study highlights the metabolic capacity of Bcc bacteria, the potential hazards posed by the presence of these opportunistic pathogens in pharmaceutical products, and the inefficacy of using sub-critical concentrations of specific biocides as a preventive measure to avoid Bcc-related contaminations. Results reinforce the urgent need to re-evaluate the application of biocides in non-sterile pharmaceutical products' formulations and the concentrations used, in order to prevent contamination and subsequent infection outbreaks. Formation of SCVs raises a serious problem for clinical microbiologists, since their reduced size and growth rate hamper detection by conventional diagnostic tests, more than often leading to false-negative results (Torbeck et al., 2011), especially when standardized incubation periods are used, which are not sufficient to allow SCV detection with the naked eye (Jimenez et al., 2015). The alterations in cellular shape that occur in response to long-term incubation under nutrient starvation may also hinder Bcc eradication from pharmaceutical products, since these particular bacterial cells might be able to pass through the membranes used during filtration processes and induce contamination (Jimenez, 2004). Bcc bacteria have a slower growth rate in comparison with other microorganisms, therefore in samples containing mixed bacterial populations, their presence might be masked by other fast-growing bacteria (Henry et al., 1999). If these bacteria are not detected during quality control, contaminated products wrongly considered as safe might reach the market and be used by patients at risk, which negatively affects their treatment options and prognosis. Bcc tolerance to BZK appears to be a multifactorial phenomenon, involving not only the induction of the genetic machinery required for the use of the biocide as a carbon and energy source, but also the emergence of different phenotypes and formation of cell aggregates rich in polysaccharide. The comparative genomic, transcriptomic and quantitative proteomic analyses of the several representative clonal morphotypes (SCVs vs. normal sized colonies and rough vs. smooth colonies) of each original bacterial population obtained during long-term incubation under nutrient starvation in saline solutions and in the presence of increasing BZK concentrations would be of utmost interest to understand the underlying mechanisms.

## Data Availability Statement

The datasets presented in this study can be found in online repositories. The names of the repository/repositories and accession number(s) can be found below: https://www.ebi.ac.uk/ena, PRJEB36038.

## Ethics Statement

Clinical isolates were recovered, as part of the hospital routine, from the sputum of CF patients under surveillance at Hospital de Santa Maria, Centro Hospitalar Lisboa Norte (CHLN) EPE. Studies involving these isolates were approved by the CHLN ethics committee and the anonymity of the patients was preserved. Informed consent was also obtained from all participants and/or their legal guardians. All the methods were performed in accordance with the relevant guidelines and regulations.

## Author Contributions

MT, MK, and AB prepared the extended incubation experiments in saline solutions supplemented with BZK and characterized the Bcc cultures during incubation. MT prepared the figures and tables and contributed to the writing of the manuscript under the scientific supervision of IS-C, who conceived and coordinated the study. VC contributed with the sequencing of genomes and also to the writing of the manuscript. CG, AH, and MT performed the genome analysis of BZK catabolic genes. All authors read and approved the final manuscript.

## Conflict of Interest

The authors declare that the research was conducted in the absence of any commercial or financial relationships that could be construed as a potential conflict of interest.
