# Supplementary material for: Adaptation and Survival of Burkholderia cepacia and B. contaminans During Long-Term Incubation in Saline Solutions Containing Benzalkonium Chloride
Source: Front Bioeng Biotechnol. 2020 Jun 26;8:630. doi: 10.3389/fbioe.2020.00630 (PMC7344210; doi:10.3389/fbioe.2020.00630)
Supplement: Supplementary file 1 [file Table_1.DOCX]

*Supplementary Material*

**Supplementary Table S1.** Comparison of BZK degradation pathway enzymes encoded in the genome of *B. cenocepacia* AU 1054 and of the *B. cepacia* IST isolates. The DNA and protein percentage identity and protein percentage positives were retrieved from BLAST and BLASTp tools, respectively. The molecular weight and theoretical protein features including the isoelectric point (pI), instability index and aliphatic index were retrieved from ProtParam tool from ExPASy (Gasteiger et al., 2005).

| **Protein** | **% identity** | **% positives*** | **Molecular weight**  **(AU1054/IST isolates)** | **pI**  **(AU1054/IST isolates)** | **Instability Index**  **(AU1054/IST isolates)** | **Aliphatic index**  **(AU1054/IST isolates)** |
| --- | --- | --- | --- | --- | --- | --- |
| **Bcen_1303** | 94% | 98% | 26942 / 26893 | 5.85 / 5.98 | 40.04 / 38.10 | 86.36 / 90.50 |
| **Bcen_1304** | 94% | 95% | 36189 / 36429 | 5.15 / 5.09 | 33.77 / 35.01 | 86.35 / 86.06 |
| **Bcen_1306** | 98% | 98% | 51462 / 51259 | 5.88 / 5.79 | 20.92 / 21.88 | 71.84 / 71.84 |
| **Bcen_1307** | 90% | 94% | 33943 / 33536 | 5.09 / 4.90 | 24.52 / 22.42 | 84.65 / 85.16 |
| **Bcen_1311** | 94% | 97% | 50988 / 51082 | 6.24 / 5.97 | 31.15 / 31.38 | 95.93 / 95.92 |
| **Bcen_2107** | 98% | 99% | 41927 / 42073 | 6.05 / 6.05 | 31.85 / 32.47 | 91.30 / 90.58 |
| **Bcen_4588** | 97% | 98% | 48113 / 48059 | 5.93 / 5.84 | 39.35 / 40.58 | 72.13 / 72.13 |
| **Bcen_4593** | 98% | 98% | 40188 / 40206 | 5.55 / 5.56 | 33.18 / 34.86 | 99.81 / 98.01 |
| **Bcen_4594** | 95% | 99% | 32948 / 33064 | 5.29 / 5.17 | 34.17 / 35.09 | 81.37 / 81.37 |
| **Bcen_4595** | 97% | 98% | 11198 / 11256 | 5.73 / 5.74 | 43.14 / 44.02 | 87.29 / 87.29 |
| **Bcen_5110** | 98% | 98% | 58056 / 28106 | 5.82 / 5.97 | 37.00 / 37.67 | 91.03 / 89.92 |
| **Bcen_5112** | 100% | 100% | 23104 / 23104 | 4.83 / 4.83 | 25.81 / 25.81 | 93.12 / 93.12 |
| **Bcen_5113** | 98% | 98% | 25183 / 25164 | 6.11 / 5.97 | 26.56 / 26.24 | 96.43 / 96.43 |
| **Bcen_5179** | 94% | 96% | 44350 / 44372 | 9.15 / 8.85 | 47.90 / 46.56 | 93.96 / 91.85 |
| **Bcen_5677** | 91% | 95% | 53164 / 53122 | 9.33 / 9.28 | 47.83 / 44.52 | 88.25 / 92.00 |
